# Unraveling the Novel Effect of Patchouli Alcohol Against the Antibiotic Resistance of *Helicobacter pylori*

**DOI:** 10.3389/fmicb.2021.674560

**Published:** 2021-06-02

**Authors:** Yuanzun Zhong, Liyao Tang, Qiuhua Deng, Li Jing, Jiao Zhang, Yao Zhang, Feng Yu, Yijun Ou, Shaoju Guo, Bin Huang, Hongying Cao, Ping Huang, Yifei Xu

**Affiliations:** ^1^School of Pharmaceutical Sciences, Guangzhou University of Chinese Medicine, Guangzhou, China; ^2^School of Basic Medical Science, Tianjin Medical University, Tianjin, China; ^3^Shenzhen Traditional Chinese Medicine Hospital, The Fourth Clinical Medical College of Guangzhou University of Chinese Medicine, Shenzhen, China

**Keywords:** *Helicobacter pylori*, patchouli alcohol, resistance, clarithromycin, intracellular

## Abstract

The long-term colonization of *Helicobacter pylori* can cause various gastrointestinal diseases, and its high genetic variability is prone to antibiotic resistance and leads to failure of clinical treatment. Intracellular survival also contributes to the drug tolerance of *H. pylori*. Patchouli alcohol (PA) shows a highly efficient activity against *H. pylori in vitro* and *in vivo*. And this study aims to explore whether PA can reduce the resistance of *H. pylori* and determine the underlying mechanism. Checkerboard and time–kill bactericidal curve assay reveal that the combination of PA and clarithromycin (CLR) promoted the inhibition and bactericidal effect against *H. pylori*. Stimulation of CLR leads to the internalization of *H. pylori*, but PA can effectively inhibit the invasion induced by CLR. Compared with antibiotics, PA remarkably eradicated the intracellular *H. pylori*, and this intracellular sterilized ability was further improved in combination with antibiotics (CLR and metronidazole). The expression of *H. pylori* efflux pump genes (*hp0605*, *hp1327*, and *hp1489*) was dose-dependently downregulated by PA. Digital droplet PCR indicated that the *H. pylori* mutant of A2143G can be inhibited by PA. Cellular uptake and transport assays showed that PA is rapidly absorbed, which promotes its activity against intracellular bacteria. Therefore, PA can act synergistically with CLR as a candidate treatment against drug-resistant *H. pylori*.

## Introduction

*Helicobacter pylori* is a Gram-negative spiral-shaped flagellated bacterium colonizing the stomach of more than half of the global population and thus considered as one of the common infecting bacteria worldwide (Salama et al., 2013; Ansari and Yamaoka, 2017; Crowe and Solomon, 2019). *H. pylori* causes various gastroduodenal diseases, such as chronic atrophic gastritis, gastric ulcer, duodenal ulcer, and gastric cancer (Choi et al., 2020; de Martel et al., 2020; Ford et al., 2020). Effective *H. pylori* eradication is beneficial for the prevention of gastric cancer (Tsukamoto et al., 2017; Liou et al., 2020). Since the 1990s, standard triple therapy comprising two antibiotics (clarithromycin [CLR], metronidazole [MTZ], or amoxicillin) and a proton pump inhibitor has been used for *H. pylori* eradication. However, the resistance of *H. pylori* to CLR and MTZ has increased due to the abuse and misuse of antibiotics. In some highly resistant areas, the resistance rates to MTZ and CLR reached 70–80 and 40–50%, respectively (Thung et al., 2016). Antibiotic resistance has reduced the eradication rate of standard triple therapy to 70% or even lower (Liu et al., 2018; De Francesco et al., 2019).

Antibiotic resistance is a global public health problem in which the sustained increase in antibiotic resistance may eventually surpass the amount of new antibiotics being developed. Bacterial resistance is a complex process between host and bacteria and may be related to the following mechanisms: mutated drug targets, enhanced efflux pump expression, altered membrane permeability, etc. (Gerrits et al., 2006; Gong and Yuan, 2018). Essentially, bacterial mutation occurs during their evolution on some key functional proteins, such as nucleic acid synthesis, redox system, and protein translation, thus contributing to antibiotic resistance (Alba et al., 2017). Alterations on the efflux system (Gerdes and Semsey, 2016) and cell membrane (Garcia-Fernandez et al., 2017) would reduce the intracellular accumulation of antibiotics, thus diminishing the antimicrobial activity. Furthermore, bacteria produce enzymes to inactivate antibiotics or release virulence factors to affect antibiotic activity (Behrens et al., 2020). The above mechanisms do not exist in isolation in the process of bacterial resistance, and recent experiments on *Escherichia coli* indicated that an increased activity of efflux pump may increase the mutation rate of bacterial resistance genes (El Meouche and Dunlop, 2018). For *H. pylori*, its cell invasion ability is one of the strategies to avoid the bactericidal effect of antibiotics (Wang et al., 2017).

clarithromycin belongs to the class of macrolide antibiotics (Vazquez-Laslop and Mankin, 2018) and exhibits its antibacterial effect by inhibiting the protein synthesis of bacteria. For some CLR-sensitive *H. pylori*, the bactericidal effect of CLR can be observed at a minimum inhibitory concentration (MIC) lower than 0.0156 μg/ml; however, for some drug-resistant strains, the anti-*H. pylori* activity is attained at an MIC of 256 μg/ml (Jung et al., 2018). Point mutations in *H. pylori* such as A2143G, A2142G, T2182C, C2611A, and T2717C (Seo et al., 2019) contribute to its CLR resistance, change the configuration of the ribosome, and weaken antibiotic binding, thereby leading to drug resistance. Among the point mutations, the A2142G and A2143G accounted for the majority (Matta et al., 2018; Marques et al., 2020).

**FIGURE 1:**
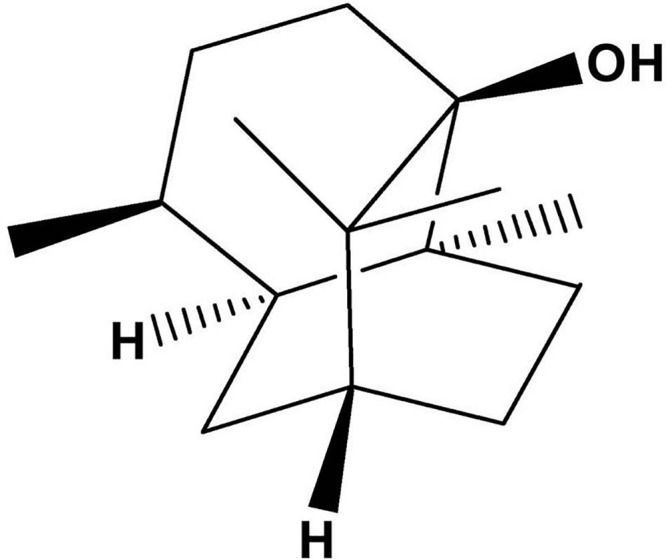
Chemical structure of PA.

Pogostemonis Herba, the dried aerial part of *Pogostemon cablin* (Blanco) Benth. (Labiatae), is a traditional Chinese medicament used to treat gastrointestinal diseases in many Asian countries. The main component of Pogostemonis Herba volatile oil is patchouli alcohol (PA, the chemical structure shown in Figure 1). Our laboratory’s previous experiment revealed that PA can effectively inhibit *H. pylori in vivo* and *in vitro* and shows a protective effect on *H. pylori*-related gastritis (Xu et al., 2017; Lian et al., 2018). A stable MIC against drug-resistant *H. pylori* and the strong post-antibiotic effect (PAE) of PA were also verified.

The objective of this study is to explore whether the combination of PA and antibiotics (CLR and MTZ) could improve the antibacterial efficiency against *H. pylori* and to investigate the underlying mechanisms.

## Materials and Methods

### Chemicals and Reagents

Columbia blood agar base, brain heart infusion (BHI), Mueller–Hinton (MH) agar, and *H. pylori* selective supplement (Dent) SR0147E were obtained from Oxoid, United Kingdom. Dulbecco’s modified Eagle’s medium (DMEM) and fetal bovine serum (FBS), Hank’s balanced salt solution (HBSS), MEM nonessential amino acids, TRIzol reagent, and Live/Dead BacLight Bacterial Viability kits (Molecular Probes) were purchased from Thermo Fisher Scientific, United States. Sheep blood was procured from Pingrui Biotechnology, China. CLR was purchased from Dalian Meilun Biotechnology, China. MTZ and saponin were acquired from Sigma, Germany. Fastking gDNA Dispelling RT SuperMix Kit, Talent qPCR PreMix (SYBR Green) Kit, TIANamp Bacteria DNA kit, and 2 × Taq PCR Mix were purchased from TIANGEN, China. Anti-*H. pylori* antibody ab20459 was obtained from Abcam, United Kingdom. Alexa Fluor 488 and goat anti-rabbit were bought from Southern Biotech, United States.

### PA Preparation

Patchouli alcohol (purity > 99%) was kindly provided by the Mathematical Engineering Academy of Chinese Medicine, and the quality of PA was confirmed by melting point, infrared spectroscopy, ^1^H and ^13^C NMR, and mass spectrometry (Liao et al., 2015). DMSO was used to dissolve PA and served as a control (DMSO < 0.5% in all experiments) in the *in vitro* study.

### *H. pylori* Strains, Cell Culture, and Growth Condition

The *H. pylori* reference strain NCTC11637 was purchased from the American Type Culture Collection (ATCC, United States). Clinical drug-resistant strains Hp1869 (MTZ-resistant strain) and Hp1870 (MTZ- and CLR-resistant strain) were obtained from Renji Hospital, Shanghai Jiao Tong University. *H. pylori* specimens were stored at −80°C in BHI containing 30% glycerol and then cultured on Columbia agar base supplemented with 5% sheep blood at 37°C in a tri-gas incubator (Nuaire Nu-5831E, United States) with 10% CO_2_, 5% O_2_, and 85% N_2_. After 48–72 h proliferation, *H. pylori* specimens were scraped, suspended in phosphate-buffered saline (PBS), and passaged to fresh Columbia blood agar. Turbidimetry (Zhuhai BaSO Biotechnology Co., Ltd., China) was measured to adjust the McFarland standard of the *H. pylori* strain when needed.

Human gastric epithelial cells GES-1 and human colon adenocarcinoma cell line Caco-2 were purchased from the BNCC Biotechnology Company (China). The GES-1 cells were cultured in high-glucose DMEM supplemented with 10% FBS, and the Caco-2 cells were cultured in DMEM containing 10% FBS, 1% nonessential amino acids, and 1% penicillin–streptomycin antibiotic solution. Both cell lines were cultured at 37°C in a 5% CO_2_ humidified incubator (CO150, United States) and passaged when they reached 80–90% confluence at a 1:2 ratio by using 0.25% trypsin.

### Checkerboard Method

The checkerboard method was used to determine the potency of the combination of PA with CLR or MTZ on *H. pylori*. The assay assesses the activities of antimicrobial combinations tested at concentrations in serial two-fold dilutions under MIC. Autoclaved MH agar (5% sheep blood) was prepared for gradient dilutions of PA, CLR, and MTZ. The final concentration of each drug ranges from 1 to 0.25 MIC; the MIC values are shown in Table 1. And according to the checkerboard method, to design different combinations of the two drugs, their combined effects must be observed. *H. pylori* was then resuspended in PBS, and the concentration was adjusted to McFarland 1 (1 × 10^8^ CFU/ml) through turbidimetry. Bacterial suspension (100 μl) was spread onto the drug-containing MH agar plates and cultured in a tri-gas incubator for 4 days. The fractional inhibitory concentration index (FICI) was calculated as the sum of the MIC of each compound used in combination and divided by the MIC of each compound used alone as follows:

**TABLE 1:** MICs for *H. pylori*^a^.

	**Agar dilution method**			**Broth dilution method**	
**Strain**	**MIC of CLR**	**MIC of MTZ**	**MIC of PA**	**MIC of CLR**	**MIC of PA**
NCTC11637	0.0156	4	25	0.0156	25
Hp1870	32	>50	25	8	25
Hp1869	0.0156	20	50	–	–

*^a^MIC is given in μg/ml.*

*“–”, the assay was not performed.*

$${\text{FIC}}_{\left(\mathrm{PA}\right)}=\left(\mathrm{MIC}\mathrm{of}\mathrm{PA}\mathrm{in}\mathrm{combination}\mathrm{with}\mathrm{antibiotic}\right)$$

/(MICofPAalone)

$${\text{FIC}}_{\left(\mathrm{antibiotic}\right)}=\left(\mathrm{MIC}\mathrm{of}\mathrm{antibiotic}\mathrm{in}\mathrm{combination}\mathrm{with}\mathrm{PA}\right)$$

/(MICofantibioticalone)

$$\text{FICI}={\text{FIC}}_{\left(\text{PA}\right)}+{\text{FIC}}_{\left(\text{antibiotic}\right)}$$

The results were interpreted using the following criteria: synergism (FICI ≤ 0.5), additive (0.5 < FICI ≤ 1), indifference (1 < FICI < 4), and antagonism (FICI ≥ 4) (Krzyzek et al., 2020).

### Bactericidal Effect of PA Combined With CLR Against *H. pylori* NCTC11637 and Hp1870

The *H. pylori* reference strain NCTC11637 and drug-resistant strain Hp1870 were used to measure the killing kinetics of PA and CLR and their combination. *H. pylori* specimens were resuspended in PBS, and the concentration was adjusted to McFarland 1 through turbidimetry. The bacteria were then mixed with BHI (10% FBS) at a ratio of 1:10 and shaken at 120 rpm in a tri-gas incubator until grown to the logarithmic growth phase (approximately 48–72 h). Different combinations of 1/2 MIC PA and 1/2 MIC CLR (the details were shown as follows) were added to explore the bactericidal effect on *H. pylori*. NCTC11637 was treated with 12.5 μg/ml PA, 0.007 μg/ml CLR, and 12.5 μg/ml PA + 0.007 μg/ml CLR, and the drug-resistant strain Hp1870 was treated with 12.5 μg/ml PA, 4 μg/ml CLR, and 12.5 μg/ml PA + 4 μg/ml CLR. The same volume of DMSO served as the control. The MIC for the liquid culture strain was tested at OD_600_ through broth dilution as shown in Table 1. Suitable samples were removed every 24 h for 0–5 days and used for the following assay.

A time–kill bactericidal curve was analyzed to confirm the synergistic activity. Approximately 100 μl of samples was removed, and the gradient dilutions were prepared and flooded on the plate to form colonies that were counted after 4 days of cultivation. The time–kill bactericidal curve was constructed by plotting log_10_ CFU/ml versus time. In time–kill bactericidal curves, synergy is defined as a ≥2-log_10_ decrease in CFU/ml between the combination and the most active constituent (Doern, 2014).

For the assay using Live/Dead BacLight Bacterial Viability kits, the samples were removed every 24 h and stained by SYTO 9 and PI (mixed at a ratio of 1:4 before use) to identify live or dead bacteria. Approximately 1 ml of the sample was centrifugated and resuspended in normal saline (NS), and a fluorescence dye was added at a ratio of 3:1,000. After incubation at room temperature in the dark for 15 min, 10 μl of the sample was diluted using 100 μl of NS in a glass-bottom dish and finally photographed by a confocal microscope (LSM 800, Zeiss).

### Gentamycin Protection Method

*Helicobacter pylori*-infected gastric epithelial cells (multiplicity of infection [MOI] = 100:1) were washed three times with PBS, followed by 100 μg/ml gentamicin incubation for 6 h to remove extracellular bacteria. Intracellular bacteria were retrieved from invaded cells by a 15 min incubation with 0.1% saponin in PBS at 37°C. Serial dilutions of bacterial suspensions were prepared using PBS and drop-plated (100 μl drop) onto Columbia blood agar plates. The bacterial colonies were counted after 4 days of cultivation in a tri-gas incubator for CFU determinations.

### Immunofluorescence Method

GES-1 cells (1.5 × 10^5^) were cultured in a glass-bottom dish, allowed to adhere for 6 h, and added with *H. pylori* NCTC11637 at an MOI of 100:1. After 12 h of co-incubation, unattached bacteria were removed by washing with PBS three times. Then, the cells were treated with 100 μg/ml gentamicin for 6 h to clear extracellular bacteria. The cells were stimulated by PA and antibiotics according to different experiment designs. Next, the cells were fixed with 4% paraformaldehyde for 30 min at room temperature and then washed three times with PBST (PBS + 0.05% Tween 20). PBST washing for three times was performed between each step below. After washing, the cells were permeabilized with PBST containing 0.1% Triton for 30 min. Goat serum (5%) containing PBST was used as a blocking buffer and incubated for 1 h. The intracellular *H. pylori* was stained by an anti-*H. pylori* antibody (1:200). The secondary antibody Alexa Fluor 488-conjugated goat anti-rabbit (1:200) was added and incubated at room temperature in the dark for 2 h. Finally, the samples were stained with DAPI, sealed, and placed on an LSM 800 confocal microscope (Carl Zeiss) for image acquisition.

### Effect of Low-Dose Antibiotics and PA on the Amount of Intracellular *H. pylori*

The GES-1 cells (1.5 × 10^6^) were seeded on a six-well plate for 6 h and then added with the *H. pylori* standard strain NCTC11637 at an MOI of 100:1. *H. pylori*-infected cells were stimulated by CLR and MTZ at 1/4 MIC and 1/8 MIC doses for 6 h, respectively. A gentamycin protection assay was conducted to measure the CFU of intracellular *H. pylori*.

*H. pylori* NCTC11637-infected GES-1 cells (MOI = 100:1) were treated by 6.25, 12.5, and 25 μM PA combined with 1/8 MIC CLR for 6 h to observe the influence of PA on *H. pylori* invasion promoted by low CLR doses. A gentamicin protection assay was used for CFU determinations, and immunofluorescence was adopted for cell imaging.

### Intracellular Bactericidal Effect of PA, CLR, MTZ, and Their Combinations

The *H. pylori* reference strain NCTC11637 and clinical drug-resistant strains Hp1869 and Hp1870 were used to compare the ability of various drugs for intracellular *H. pylori* elimination. The GES-1 cells (1.5 × 10^6^) were seeded on a six-well plate, allowed to adhere for 6 h, and then added with the bacteria at an MOI of 100:1.

The killing ability for intracellular *H. pylori* was investigated by using PA, MTZ, or CLR alone. After extracellular bacteria were eliminated by gentamicin for 6 h, the *H. pylori* -infected GES-1 cells were treated with CLR (0.0156, 0.0312, 0.078, 0.156, and 0.312 μg/ml for NCTC11637 and Hp1869; 16, 32, 48, 64, and 72 μg/ml for Hp1870), MTZ (12.5, 25, 50, 75, and 100 μg/ml for all three strains), and PA (25, 30, 35, 40, 45, and 50 μg/ml for all three strains) for 16 h. Intracellular *H. pylori* survival was examined as described in the gentamycin protection assay.

The combined effects of CLR, MTZ, and PA on intracellular bactericidal effect were further explored. After extracellular bacteria were eliminated by gentamycin for 6 h, the infected GES-1 cells were further incubated with 25 μg/ml PA in combination with antibiotics for 16 h. The doses of CLR were 0.0312, 0.078, and 0.156 μg/ml for NCTC11637 and Hp1869 and 32, 48, and 64 μg/ml for Hp1870. For MTZ, the doses were selected as 25, 50, and 75 μg/ml. Meanwhile, to further study the effect of PA, the combination of CLR and MTZ has also been tested. For NCTC11637 and Hp1869, 0.015 μg/ml CLR combined with 75 μg/ml MTZ or 0.15 μg/ml CLR combined with 25 μg/ml MTZ was used for 16 h. For Hp1870, 32 μg/ml CLR combined with 75 μg/ml MTZ or 64 μg/ml CLR combined with 25 μg/ml MTZ was used. Finally, intracellular *H. pylori* survival was analyzed as described in the gentamycin protection assay. Relative to the control group, a 99% decrease in the viability of intracellular strains was defined as effective extermination.

Immunofluorescence was used to compare the number of residual intracellular *H. pylori*. After extracellular bacteria were eliminated by gentamicin, NCTC11637-infected GES-1 cells were treated by CLR (0.312 μg/ml, 20 MIC), MTZ (100 μg/ml, 25 MIC), and PA (50 μg/ml, 2 MIC) for 16 h. The cells were imaged using the immunofluorescence method.

### Efflux Effect Gene Expression and PA’s Influence

The multidrug-resistant *H. pylori* strain Hp1870 was used to observe the effect of PA on the expression of the efflux effect gene. Hp1870 with concentration adjusted to 1 × 10^8^ CFU/ml through turbidimetry was cultured, mixed with BHI (10% FBS) at a ratio of 1:10, and classified into a negative control group (DMSO), model group (32 μg/ml CLR), and PA + CLR treatment groups (12.5, 25, and 50 + 32 μg/ml CLR). The bacteria were stimulated for 2 h in each group. An RT-qPCR assay was adopted to measure the gene expression of efflux effect genes as described below.

The time- and dose-dependent effects of PA on the altered expression of *hp0605* induced by CLR were explored. Hp1870 specimens were treated with DMSO (negative control), 16 or 32 μg/ml CLR (1/2 MIC or MIC, model group), or CLR + PA (16 or 32 μg/ml CLR + 50 μg/ml PA) for 0.5 or 2 h. An RT-qPCR assay was adopted to measure the *hp0605* expression.

After the above stimulation, a TRIzol reagent was used to extract bacterial total RNA. RNA purity and concentration were determined based on A260/280 and A260, respectively, by using a NanoDrop 2000 UV–vis spectrophotometer. The total RNA was reverse transcribed into cDNA using the Fastking gDNA Dispelling RT SuperMix Kit. RT-qPCR was performed using the CFX96 (Bio-Rad, United States) for one cycle at 95°C for 3 min and 40 cycles at 95°C for 5 s and 60°C for 15 s. Data were analyzed using the Pfaffli method with the *16s* gene as the internal reference. The primers used in RT-qPCR are shown in Table 2.

**TABLE 2:** Primer sequences for qPCR and PCR.

**Gene**	**Forward primer (5′–3′)**	**Reverse primer (5′–3′)**
*hp0605*	AGCGCAAGAACTCAGTGTCA	GCTTGGAGTTGTTGGGTGTT
*hp1327*	GCCAGGCTTGATGAAGAAAA	TTAGCCTGCTTGCCGTAAAT
*hp1489*	TAGGCGCTCAAGTGGCTTAT	TCAGATCGGGCAGATTTTTC
*16S*	CGATGGATGCTAGTTGTTGGAG	GTCCCCGTCTATTCCTTTGAGTT
*23S*	GTAACTATAACGGTCCTAAG	GAAACATCAAGGGTGGTATC

### Droplet Digital PCR

The multidrug-resistant *H. pylori* strain Hp1870 was cultured, and its concentration was adjusted to McFarland 1 using PBS. The bacterial suspension was then added to BHI (10% FBS) at a ratio of 1:10. Five groups were established in this experiment: negative control (DMSO), model group (32 μg/ml CLR), and PA + CLR groups (12.5, 25, and 50 μg/ml + 32 μg/ml CLR). The bacteria were treated for 2 h, and the bacterial total RNA was extracted by a TRIzol reagent and reverse transcribed into cDNA as described above.

PCR experiments were conducted using the 2 × Taq PCR Mix to identify the mutation site of Hp1870 by amplifying and sequencing *H. pylori 23S* genes. The primers are shown in Table 2. *H. pylori* -specific droplet digital PCR (ddPCR) assays were performed using a QX200 ddPCR system (Bio-Rad, United States) by Sangon Biotech (China). The primer and probe sequences for the CLR-resistant *H. pylori 23S* ddPCR assay are listed in Table 3 and quoted from an existing study (Sun et al., 2018). The thermal cycling conditions were as follows: 95°C for 10 min, 40 cycles of 94°C for 30 s and 60°C for 1 min, and one cycle of 98°C for 10 min. Fluorescent amplitudes were analyzed by using the QX200 Droplet Reader (Bio-Rad). Data analysis was performed by using the QuantaSoft software version 1.6.6 (Bio-Rad).

**TABLE 3:** Primers and probes used for the ddPCR assay.

**Primer or probe name**	**Sequence and chemical modification(s)^a^**
Hp23SF	5′-TCCCGTTAGCAGTGCTAA-3′
Hp23SR	5′-AGATGGGAGCTGTCTCAAC-3′
HP23S_WT_HEX	5′-HEX-AAGACGGAAAGACCCCGTG-BHQ1-3′
HP23S_A2143G_FAM	5′-FAM-AAGACGGAGAGACCCCGT-BHQ1-3′

*^*a*^HEX, hexachlorofluorescein; FAM, 6-carboxyfluorescein; BHQ1, Black Hole quencher 1.*

### GC–MS

GC–MS was performed on an Agilent 7890A-5975C GC–MS system (Agilent, United States). GC separation was conducted on an HP-5MS capillary column (30 m × 0.25 mm, 0.25 μm). The initial oven temperature was set at 160°C and then programmed to increase to 220°C by a gradient of 6°C/min. The inlet temperature was 230°C. MS conditions were as follows: EI mode, ionization energy 70 eV, ionization source temperature 250°C. The parent ion and product ion mass spectra of the base ion peaks at 222.2 and 150.2 *m*/*z* for PA and internal standard cedrol, respectively, were acquired with selected ion monitoring. The split ratio was 1:2, and the inject volume was 1 μl.

### Cellular Uptake Assay

For cellular uptake experiments, the Caco-2 cells were seeded onto 12-well plates at 1 × 10^5^ cells per well, grown for 14 days, washed with HBSS (37°C) twice, and incubated with 1 ml of 300, 500, and 700 μM PA in HBSS (pH 4.1, 7.4, or 9.1) for 5–30 min in 37°C or 4°C. After incubation, the cells were washed with HBSS (4°C) three times. Next, the PA within the cells was extracted by ultrasound in 1 ml of cell lysis buffer (HBSS containing 10% methanol). After centrifugation for 10 min at 8,000 *g*, the supernatant was removed and divided into two parts; one was used to determine the protein concentration by a BCA kit, and the other was used to determine PA concentration by GC–MS after extraction with ethyl acetate.

### Cellular Transport Assay

For cellular transport experiments, the Caco-2 cells were seeded onto polycarbonate filter membranes (pore size 0.4 μm, filter area 1.12 cm^2^) in 12-well plates at 1 × 10^5^ cells per well and grown for 21 days. Trans-epithelial electrical resistance (TEER) values were measured twice weekly by using a Millicell-ERS volt-ohm meter, and the membranes with monolayer cells that met the criteria with a TEER of above 500 Ω/cm^2^ were used for the transport studies. Caco-2 cell monolayers were washed twice with HBSS (pH 7.4), and PA (300, 500, and 700 μM) was added to either the apical (AP, 0.5 ml) or basolateral (BL, 1 ml) side. The receiving chamber contained the corresponding volume of HBSS. Verapamil (100 μM) was used to investigate the role of p-glycoprotein (P-gp) in PA absorption. After shaking at 54 rpm for 2 h, the samples were collected from the receiving chamber to assess the drug transport from the AP-to-BL or BL-to-AP direction. The PA content was measured by GC–MS after extraction with ethyl acetate. The apparent permeability coefficient (*Papp*) was calculated from the following equation.

$$Papp\left(\frac{cm}{s}\right)=\frac{\Delta Q}{\Delta t\times A\times C_{0}},$$

where Δ*Q/*Δ*t* is the cumulative transport rate of the compound on the receiving side (μM/s), *A* is the surface area of the cell monolayer (cm^2^), and *C*_0_ is the initial concentration in the donor compartment (μM/cm).

### Statistical Analysis

All results were presented as means ± standard deviation and analyzed using ANOVA and unpaired Student *t* test in SPSS 24.0. Bonferroni’s test or Dunnett’s test was performed for multiple groups based on the homogeneity of variance to test the significant difference. A *t*-test was performed for two independent groups. *P* < 0.05 was considered significant.

## Results

### Combined Anti-*H. pylori* Effect of PA and Antibiotics (CLR and MTZ)

**FIGURE 2:**
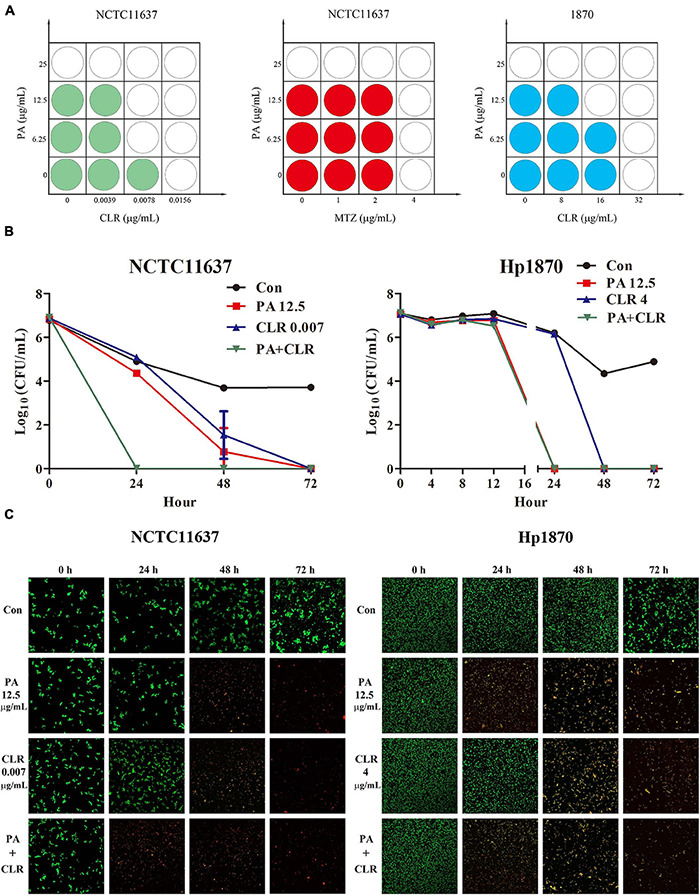
Combined effect of PA and antibiotics against *H. pylori*. **(A)** Combined effect tested by the checkerboard method of PA and antibiotics against the *H. pylori* standard strain NCTC11637 and the drug-resistant strain Hp1870 as determined by FICI (*n* = 4). **(B)** Combined effect tested by time–kill curves of PA and CLR against the *H. pylori* strains NCTC11637 and Hp1870; the synergy is defined as a ≥2-log_10_ (*n* = 3). **(C)** Bacteria imaging by Live/Dead BacLight Bacterial Viability kits to observe the bactericidal capacity of PA and CLR against *H. pylori* NCTC11637 and Hp1870.

The effects of PA combined with CLR and MTZ on the *H. pylori* reference strain NCTC11637 and the clinical strain Hp1870 (CLR and MTZ resistant) were observed using the checkerboard method (Figure 2A). For the reference strain NCTC11637, the CLR-and-PA combination decreased the MIC of PA from 25 to 6.25 μg/ml (FIC = 0.25) and the MIC of CLR by half (FIC = 0.5). No alteration was induced by the PA-and-MTZ combination (FIC = 1). For the clinical resistant strain Hp1870, the MIC values were reduced by half when PA was combined with CLR or vice versa (FIC = 0.5). For PA combined with CLR, the FICI was 0.75 for the reference strain NCTC11637 and 1 for the clinical resistant strain Hp1870, indicating that both components had an additive effect. For PA combined with MTZ, the FICI was 2 for the reference strain NCTC11637, which is considered as indifferent.

The time–kill bactericidal curve method was adopted to explore the combined efficiency of PA and CLR, and the results are shown in Figure 2B. For the reference strain NCTC11637, PA and CLR alone would eradicate the bacteria in 72 h. Their combination can effectively kill the bacteria in 24 h, indicating successful synergy.

For the drug-resistant strain Hp1870, the number of bacteria in each group did not change significantly after the treatment for 8 h. The number of bacteria in the group treated with PA combined with CLR decreased by approximately 75% compared with that in the control group after 12 h, indicating the faster antibacterial action of this treatment compared with drug use alone. Treatment with PA alone or PA combined with CLR killed *H. pylori* within 24 h. However, the time had extended to 48 h when the bacteria were treated with CLR only. Figure 2C displays the visible alteration of bacterial death in a time- and dose-dependent manner and corresponds to the results of the time–kill curve.

### Influence of Low-Dose CLR and MTZ on the Number of Invasive *H. pylori* and the Effect of PA

**FIGURE 3:**
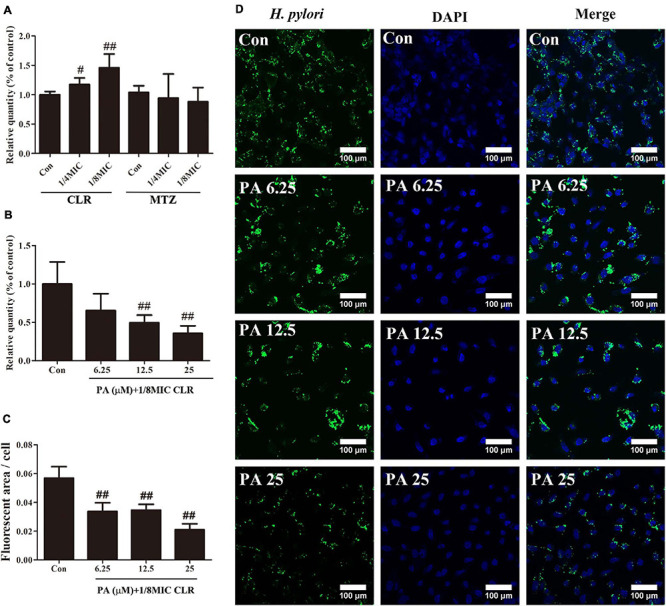
Number of intracellular *H. pylori* stimulated by antibiotics and PA. **(A)** Influence of low-dose CLR and MTZ on the number of intracellular *H. pylori*. **(B)** Antagonism effect of PA on the increased invasion induced by CLR; a gentamycin protection assay was conducted to measure the CFU of intracellular *H. pylori*. **(C)** Quantitative results of the fluorescent area of *H. pylori* (green area) with treatment of PA and CLR. **(D)** Representative confocal images of intracellular *H. pylori* after treatment with PA and CLR. The above results are expressed as the mean ± SD (*n* = 6). **^#^***P* < 0.05, and ^##^*P* < 0.01 (compared with the results for the control group).

Figure 3A shows that after 6 h of stimulation by CLR (1/8 MIC), the amount of *H. pylori* NCTC11637 that invaded into the cells were significantly increased in a concentration-dependent manner compared with that in the control group. For MTZ, no remarkable difference from the control group was observed after 6 h of stimulation.

Figure 3B indicates that PA addition effectively and concentration-dependently reduced the increment of the number of invasive *H. pylori* caused by CLR. Immunofluorescence images also reflected the same trend. Compared with that in the control group, the fluorescent area of *H. pylori* (green area) was significantly decreased by the PA treatment in different concentrations (Figures 3C,D).

### Effect of PA, CLR, and MTZ on Intracellular *H. pylori* Elimination

**FIGURE 4:**
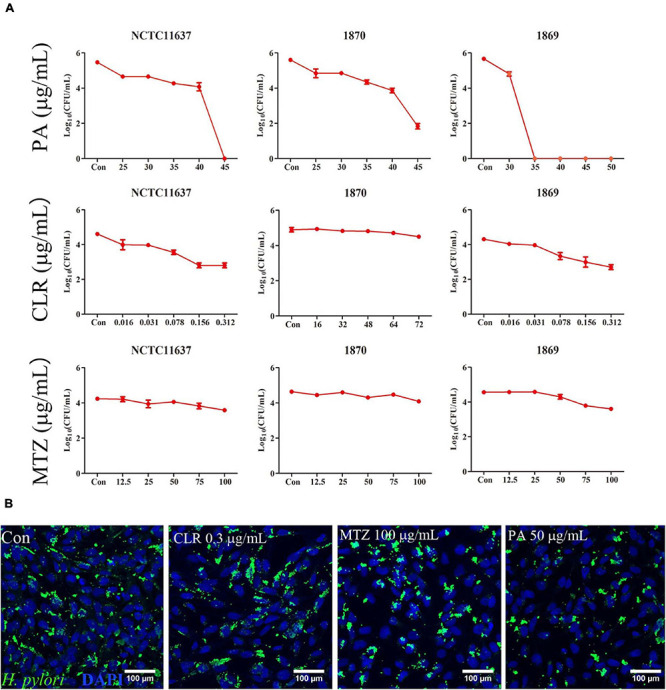
Killing ability for intracellular *H. pylori*. **(A)** Intracellular *H. pylori* standard strain NCTC11637 and drug-resistant strains Hp1869 and Hp1870 were treated with different doses of PA, CLR, and MTZ, and the number of bacteria was presented as CFU/ml (*n* = 3). Compared with the control group, a 99% decrease in the intracellular strain viability was defined as an effective extermination. **(B)** Representative confocal images by the immunofluorescence method to observe the ability of PA (2 MIC), CLR (20 MIC), and MTZ (25 MIC) to kill intracellular *H. pylori* NCTC11637; the bacteria were stained by anti- *H. pylori* antibody ab20459 (green).

As shown in Figure 4A, PA at a dose of 45 μg/ml (1.8 MIC) effectively eliminated the intracellular *H. pylori* NCTC11637 and Hp1870, and this dose was reduced to 35 μg/ml for Hp1869 (1.4 MIC). CLR at a dose of 0.156 (10 MIC)–0.312 (20 MIC) showed slight intracellular *H. pylori* elimination ability for NCTC11637 and Hp1869 strains, which are sensitive to CLR; however, the efficiencies did not reach 99% (two order of magnitude decrease in log_10_ CFU/ml). For the CLR- and MTZ-resistant strain Hp1870, no alteration was noted even when the CLR dose reached 72 μg/ml. Various MTZ doses also did not induce any changes on these three intracellular strains.

Immunofluorescence was used for visualization to compare the killing ability of CLR, MTZ, and PA for intracellular the *H. pylori* strain NCTC11637 as shown in Figure 4B. Compared with that in the control group, the reduction of intracellular *H. pylori* (green area) was observed in 50 μg/ml PA. No significant changes were found in the other groups (0.3 μg/ml CLR and 100 μg/ml MTZ).

### Ability of PA Combined With Antibiotics to Kill Intracellular *H. pylori*

**FIGURE 5:**
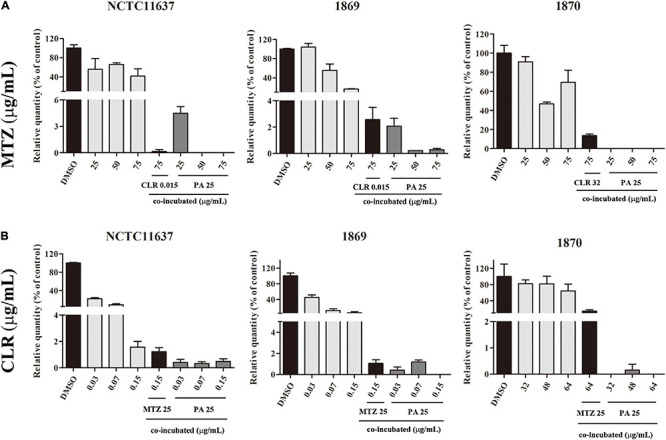
Combined effect of PA and antibiotics against intracellular *H. pylori*. **(A)** Killing ability of MTZ alone and in combination with PA (25 μg/ml) against the intracellular *H. pylori* standard strain NCTC11637 and drug-resistant strains Hp1869 and Hp1870 (*n* = 3). **(B)** Killing ability of CLR alone and in combination with PA (25 μg/ml) against intracellular the *H. pylori* standard strain NCTC11637 and drug-resistant strains Hp1869 and Hp1870 (*n* = 3). Compared with the control group, a 99% decrease in the intracellular strain viability was defined as an effective extermination.

As shown in Figure 5A, intracellular *H. pylori* strains NCTC11637, Hp1869, and Hp1870 can be effectively eliminated by 50 and 75 μg/ml MTZ combined with 25 μg/ml PA. However, 25 μg/ml MTZ combined with 25 μg/ml PA can only effectively eliminate intracellular Hp1870.

As shown in Figure 5B, the killing ability of CLR combined with 25 μg/ml PA for intracellular *H. pylori* was enhanced for the standard strain NCTC11637 and drug-resistant strains Hp1869 and Hp1870 at multiple dosages.

For comparison, the combined use of the two antibiotics showed killing ability against the standard strain NCTC11637. For drug-resistant intracellular *H. pylori*, except for high-dose CLR combined with MTZ, which is effective for Hp1869, no effective killing was achieved in other cases.

### Influence of PA on Efflux Pump Genes’ Expression in the CLR-Resistant Stain Hp1870

**FIGURE 6:**
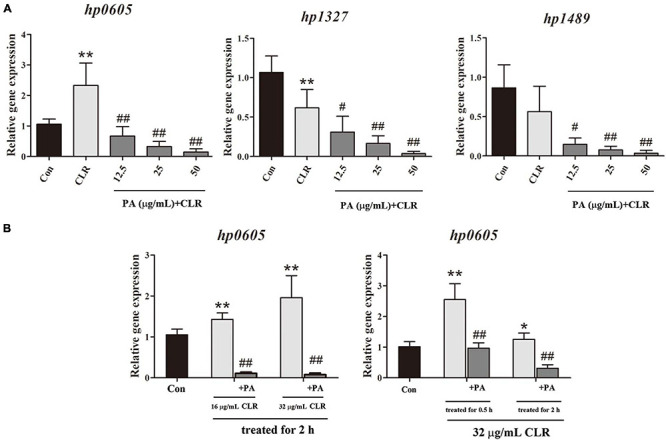
Expression of efflux effect gene. **(A)** Expression of different efflux effect homolog genes of Hp1870 after being incubated with DMSO, CLR, and PA. **(B)** Expression of *hp0605* after being stimulated with different doses of CLR and PA for different times. The results are expressed as the mean ± SD (*n* = 6). *P < 0.05 and **P < 0.01, compared with the results for the control group; **^#^***P* < 0.05 and ^##^*P* < 0.01, compared with the results for the model group.

The expression of each efflux pump gene in Hp1870 is shown in Figure 6A. The expression of *hp0605* was significantly increased, whereas that of *hp1327* was decreased with 32 μg/ml CLR treatment. PA addition at 12.5, 25, and 50 μg/ml significantly and concentration-dependently downregulated the expression of efflux pump genes including *hp0605*, *hp1327*, and *hp1489*.

As shown in Figure 6B, the effects of CLR on *hp0605* expression at different times and doses were also investigated. After stimulation with the MIC of CLR for 0.5 and 2 h, the *hp0605* expression was increased remarkably. In the dose-dependent experiment, 1/2 MIC and MIC of CLR were adopted to stimulate the bacteria for 2 h. The *hp0605* expression in CLR groups was higher than that in the control group in a dose-dependent manner and was significantly reduced with PA treatment regardless of times or doses of the CLR stimulation.

### The Rate of Mutation A2143G Expression in *H. pylori* Hp1870

**FIGURE 7:**
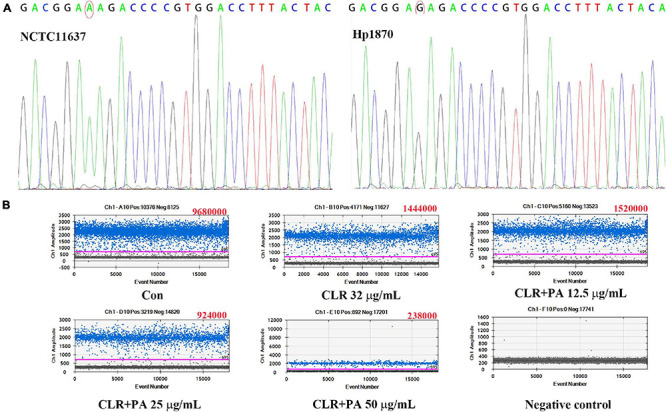
Determination of the mutation site and expression of the mutant gene in the drug-resistant strain Hp1870. **(A)** First-generation sequencing to determine the mutation of Hp1870. **(B)** Copy number of A2143G after being treated with DMSO, CLR, and PA. The blue droplets were droplets positive for A2143G, and the copy number value is shown in the upper right corner of each figure.

The sequencing results of NCTC11637 and Hp1870 indicated that the drug-resistant *H. pylori* strain Hp1870 is a CLR A2143G mutant strain (Figure 7A). ddPCR was used to detect the rate of mutation of A2143G in each group, and the results are shown in Figure 7B. PA at 25 and 50 μg/ml combined with the MIC of CLR reduced the mutation rate of 23S rRNA in the A2143G position in Hp1870.

### Uptake and Transport of PA in Caco-2 Monolayers

**FIGURE 8:**
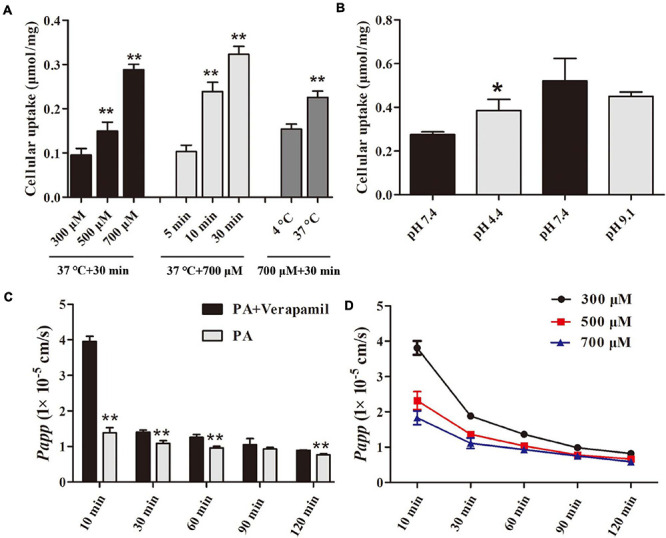
Uptake and transport of PA in Caco-2 monolayers. **(A, B)** Effect of concentrations, time, temperature, and pH on uptake of PA by Caco-2 cells. **(C, D)** Effect of time, efflux transporter inhibitor, and concentrations on transport of PA by Caco-2 cells. The data were expressed as the mean ± SD (*n* = 4). *P < 0.05 and **P < 0.01, compared with control group.

The methodology of cellular uptake and transport assay was examined, and the results were presented in the Supplementary Materials. Figure 8A indicates that the uptake of PA by Caco-2 cells increased with concentration, time, and temperature and was significantly increased in acidic conditions but not in alkaline conditions as shown in Figure 8B. Table 4 and Figures 8C,D suggested that PA is rapidly absorbed, and its main absorption mechanism is possibly passive transport.

**TABLE 4:** The effect of time on transport of PA in the Caco-2 cell monolayer model ($\bar{x}$ ± SD, *n* = 3).

**Time (min)**	***Papp* (10^–5^ cm/s)**		
	**AP → BL**	**BL → AP**	**Efflux ratio**
10	1.73 ± 0.513	1.11 ± 0.087	0.64
30	1.70 ± 0.041	0.97 ± 0.069	0.57
60	1.06 ± 0.042	0.86 ± 0.040	0.81
90	1.30 ± 0.018	0.83 ± 0.036	0.64
120	0.98 ± 0.011	0.68 ± 0.031	0.70

## Discussion

Since antibiotics have been discovered and used to treat infectious diseases in the 20th century, many diseases caused by bacterial infections have been effectively controlled. However, with the indiscriminate use of antibiotics, bacteria developed tolerance to antibiotics and then drug resistance (Levin-Reisman et al., 2017). Bacterial resistance has become a global health issue, which can be solved by developing new antimicrobial agents or reducing bacterial resistance (Alekshun and Levy, 2007). As a commonly resistant specie, *H. pylori* has a high degree of genetic variability and is prone to produce antibiotic-resistant genes (Wang et al., 2005). This species has a strong resistance to various antibiotics, which often leads to the failure of eradication. Hence, patients have to undergo multiple subsequent treatments, thus increasing the cost and the duration of treatment (Fallone et al., 2019). When the first treatment fails, the strain in the patient is prone to develop resistance after exposure to antibiotics, leading to the increased difficulty of follow-up treatments (Lee et al., 2019).

Common drug-resistant antibiotics of *H. pylori* include MTZ and CLR. *In vitro* checkerboard method and time–kill curve experiments showed that a combination with PA can decrease the MIC and enhance the bactericidal efficacy of CLR. These results suggest that PA may increase the sensitivity of *H. pylori* to CLR. Some studies have suggested that FICI can show synergistic or additive effects when combined with drugs that have the same antibacterial or bactericidal mechanisms (Abstract Culp et al., 2020). Therefore, PA may disturb the protein synthesis of *H. pylori* or interfere with the resistant mechanism of CLR resistance to *H. pylori*. The influence of PA on A2143G could be the mechanism improving the sensibility of *H. pylori* to CLR.

In clinical practice, antibiotic dosage selection should consider efficacy and patient compliance (Suzuki et al., 2020). According to the experiments with low doses of antibiotics to stimulate *H. pylori*, the use of inappropriate doses of CLR to eliminate *H. pylori* will increase the number of intracellular *H. pylori*. Consequently, the usage of CLR should be considered cautiously in clinical treatment. The cellular internalization of *H. pylori* is one of the strategies for its drug resistance development (Capurro et al., 2019). When PA was combined with CLR for treatment, the amount of *H. pylori* invading into the cells could be effectively reduced. Additional experiments should be conducted to determine the mechanism. Follow-up experiments should try more antibiotics to summarize the relationship between bacterial internalization and antibiotics.

*H. pylori* is generally an extracellular, noninvasive bacterium (Capurro et al., 2020); however, evidence suggests that it can alter the tight cellular connections between gastric epithelial cells through complex processes, allowing it to hide in an intracellular niche for protection from antibiotic eradication therapy and thus endowing it with persistence and recolonization. Gentamicin protection experiments showed that several treatments of MIC doses of CLR and MTZ did not have sufficient ability to kill intracellular *H. pylori* (Sisto et al., 2016). However, the intracellular *H. pylori* can be effectively killed by PA in low MIC. When the antibiotic is combined with PA, it showed a bactericidal ability against intracellular *H. pylori*. In the assay of killing intracellular *H. pylori*, the maximum antibiotic dose was determined based on the antibiotic solubility and cytotoxicity. Although the immunofluorescence method provided visualized images, the anti- *H. pylori* antibody did not distinguish between dead and live bacteria, and some dead adhered *H. pylori* cannot be washed off by PBS. Therefore, a small number of dead bacteria were still stained with fluorescence, leading to inconsistent results of gentamycin protection assay.

The efflux pump of bacteria is one of the main mechanisms of bacterial resistance (Bergmiller et al., 2017; Nolivos et al., 2019). Studies on *E. coli* efflux pumps proved that the RND efflux pumps of Gram-negative bacteria mainly belong to the TolC-AcrAB and OprM-MexAB systems (Li et al., 2015). *H. pylori* has four TolC-AcrAB homologs (*HP0605-HP0607*, *HP0971-HP0969*, *HP1327-HP1329*, and *HP1489-HP1487*) (van Amsterdam et al., 2005). With the stimulation of CLR in MIC dose, the efflux pump genes were significant in Hp1870 but not in NCTC11637, which suggests that the efflux pump plays an important role in drug-resistant *H. pylori* strains. In Hp1870, the expression of *hp0605* was significantly increased, whereas those of other homologue genes were decreased. This phenomenon may be related to the main role of the *HP0605-HP0607* homolog in Asian *H. pylori* strains (Monteiro et al., 2000). Nonetheless, the expression of each efflux pump gene was decreased when PA was used. In addition, the expression of the A2143G mutant gene was also decreased by PA treatment. In further research, CLR was used to induce A2143G mutation in the standard strain NCTC11637 to observe the effect of PA on the resistant mutation rate. However, the mutant strain was not successfully produced, and the related mechanism and research need to be further clarified.

The Caco-2 monolayer is applied to preclinical investigations to predict the gastrointestinal permeability of drugs because it expresses transporters, microvilli, and enterocytes of identical characteristics to humans (Ponce de Leon-Rodriguez et al., 2019). The FDA has recommended this model as an integral component of the Biopharmaceutics Classification System (BCS). Therefore, the Caco-2 cell line was chosen as a model to explore the uptake and transport of PA. Verapamil is a classical P-gp transporter inhibitor (Aller et al., 2009). In the verapamil-related transport experiment, the *Papp* of PA was increased with verapamil treatment, indicating that the P-gp efflux transporter contributes to PA absorption. PA was rapidly absorbed by the cells, which is beneficial for killing intracellular *H. pylori*. In addition, the rapid absorption rate of PA was observed in acidic conditions, suggesting that the acidic condition in the stomach could promote the permeability of human cells to PA.

On the basis of the above results and the common drug resistance mechanism of *H. pylori*, the combination of PA and CLR showed a highly efficient bactericidal ability against *H. pylori* whether *in vitro* or *in vivo*. The inhibited expression of the efflux pump gene, A2143G mutation, and high PA absorption by cells contributed to the combined effect. The results reveal that *H. pylori* invasion is promoted by the inappropriate use of CLR and thus must be considered in clinical treatment. In conclusion, PA can act additively with CLR against *H. pylori*, indicating its potential as a candidate medication for drug-resistant *H. pylori*.

## Data Availability Statement

The raw data supporting the conclusions of this article will be made available by the authors, without undue reservation.

## Author Contributions

YZ, LT, and QD performed the experiments and wrote the manuscript. LJ revised the manuscript. JZ and YZ summarized and analyzed the data. FY and YO carried out the anti-intracellular *H. pylori* assay. SG and BH were responsible for the management and coordination of the research activity planning and execution. HC, PH, and YX designed the study and finally revised the manuscript. All authors contributed to the article and approved the submitted version.

## Conflict of Interest

The authors declare that the research was conducted in the absence of any commercial or financial relationships that could be construed as a potential conflict of interest.
