# Supplementary material for: Unraveling the Novel Effect of Patchouli Alcohol Against the Antibiotic Resistance of Helicobacter pylori
Source: Front Microbiol. 2021 Jun 2;12:674560. doi: 10.3389/fmicb.2021.674560 (PMC8206506; doi:10.3389/fmicb.2021.674560)
Supplement: Supplementary file 1 [file Data_Sheet_1.PDF]

SUPPLEMENTS

Figure S1 was the results of the expression of efflux pump genes after PA treatment. Figure S2, Table S1 and S2 were the results of the methodology of cellular uptake experiments. And Figure S3, Table S3 and S4 were the results of the methodology of cellular transport experiments. In these two cell models, two simple and selective GC/MS methods for the quantification of PA were developed and validated.

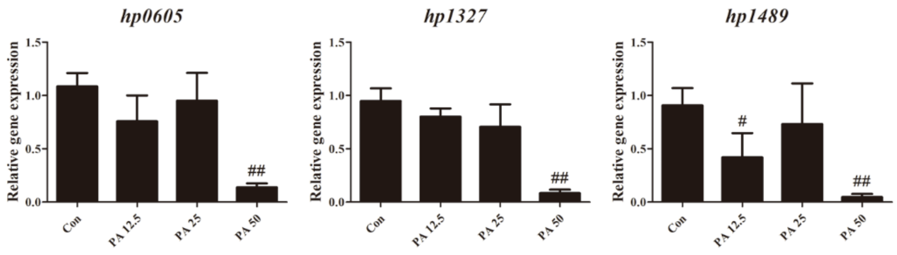

Fig. S1 The expression of efflux pump genes after PA treatment

The drug-resistant strain Hp1870 is treated with different concentration of PA, and the results are shown in the figure below. The expression of efflux pump gene did not change significantly after 12.5 and 25 μg/mL PA stimulation. But the expression of related efflux pump genes can be significantly inhibited by 50 μg/mL PA. The relevant experimental results have been added in the supplements.

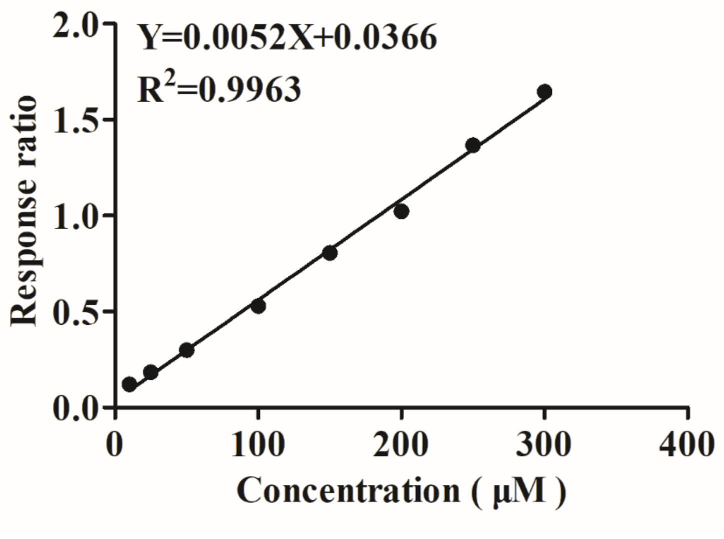

Fig. S2 Standard curve of uptake experiments

Table S1 Intra-day and inter-day precision and recovery of PA in the HBSS solution (with 10% MeOH), $\bar{x}$±s (n = 5)

Concentration (μM)	Precision (RSD%)		Recovery	
	Intra-day	Inter-day	Mean±SD (%)	RSD (%)
10	4.12	2.28	99.09	4.36
150	1.67	1.20	93.06	2.19
300	0.84	0.68	99.60	0.94

Table S2 Stability of PA at 3 QCs (n = 5)

Concentration (μM)	Intra-day	Inter-day
	RSD (%)	RSD (%)
10	4.12	4.68
150	1.67	2.30
300	0.84	1.34

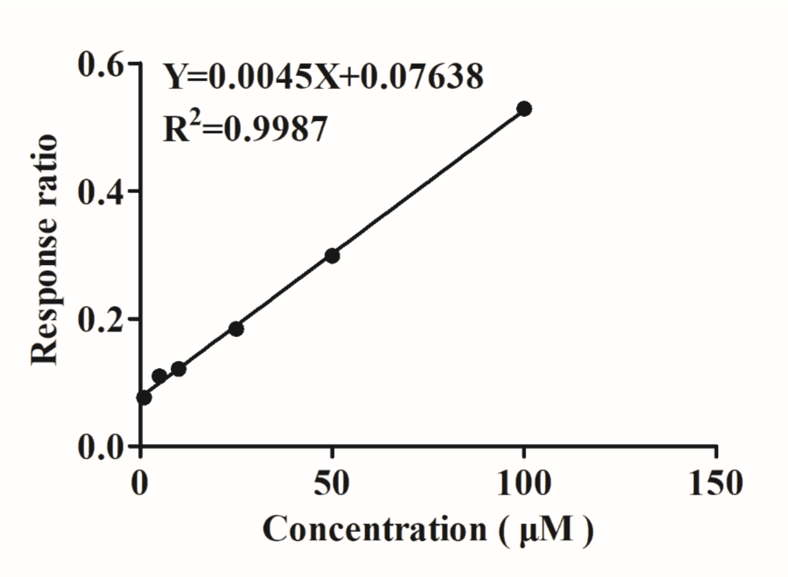

Fig. S3 Standard curve of transport experiments

Table S3 Intra-day and inter-day precision and recovery of PA in the HBSS solution, $\bar{x}$±s (n = 5)

Concentration (μM)	Precision (RSD%)		Recovery	
	Intra-day	Inter-day	Mean±SD (%)	RSD (%)
1	8.56	5.34	91.63	2.48
10	4.12	4.01	101.94	4.99
50	2.41	1.86	98.01	0.98

Table S4 Stability of PA at 3 QCs (n = 5)

Concentration (μM)	Intra-day	Inter-day
	RSD (%)	RSD (%)
1	3.99	5.39
10	5.31	4.68
50	1.09	1.53
